# Malonic Acid Isolated from *Pinus densiflora* Inhibits UVB-Induced Oxidative Stress and Inflammation in HaCaT Keratinocytes

**DOI:** 10.3390/polym13050816

**Published:** 2021-03-07

**Authors:** Cheolwoo Park, Jaeyoung Park, Won-Jin Kim, Woong Kim, Hyeonsook Cheong, Seok-Jun Kim

**Affiliations:** 1 The Garden of Natural Solution, Osan-si, Gyeonggi-do 18103, Korea; cwpark@naturalsolution.co.kr; 2 HealthMED Co.Ltd., Gwangju 61021, Korea; vanadium@hanmail.net (J.P.); hscheong@chosun.ac.kr (H.C.); 3 Department of Integrative Biological Sciences & BK21 FOUR Educational Research Group for Age-Associated Disorder Control Technology, Chosun University, Gwangju 61452, Korea; 20120624@chosun.kr (W.-J.K.); gadak2@chosun.kr (W.K.); 4 Department of Biomedical Science, Chosun University, Gwangju 61452, Korea

**Keywords:** *Pinus densiflora*, malonic acid, UVB, oxidative stress, inflammation

## Abstract

Skin aging is caused by exposure to various external factors. Ultraviolet B (UVB) irradiation induces oxidative stress, photoaging, and inflammation in skin cells. *Pinus densiflora Sieb. et Zucc.* (red pine) has various antimicrobial and antioxidant activities. However, the anti-inflammatory effects of red pine on skin have rarely been reported. The protective effects of malonic acid (MA) isolated from *Pinus densiflora* were investigated against UVB-induced damage in an immortalized human keratinocyte cell line (HaCaT). MA increased levels of the antioxidant enzymes superoxide dismutase 1 (SOD-1) and heme oxygenase 1 (HO-1) via activation of nuclear factor-erythroid 2-related factor-2 (Nrf2), resulting in a reduction in UVB-induced reactive oxygen species (ROS) levels. Additionally, the inhibition of ROS increased HaCaT cell survival rate. Thus, MA downregulated the expression of ROS-induced nuclear factor-κB, as well as inflammation-related cytokines (interleukin-6, cyclooxygenase-2, and tumor necrosis factor-α). Furthermore, MA significantly suppressed the mitogen-activated protein kinase/activator protein 1 signaling pathway and reduced the expression of matrix metalloproteinases (MMPs; MMP-1, MMP-3, and MMP-9). In contrast, MA treatment increased the expression of collagen synthesis regulatory genes (COL1A1 and COL3A1) via regulation of Smad2/3 signal induction through transforming growth factor-β. In conclusion, MA protected against UVB-induced photoaging via suppression of skin inflammation and induction of collagen biosynthesis.

## 1. Introduction

Skin aging is caused by exposure to various external factors, such as ultraviolet (UV) radiation, chemicals, and physical stimulation [1]. Excessive exposure to ultraviolet B (UVB) radiation causes erythema, edema, sunburn, hyperplasia, inflammation, immunosuppression, skin photoaging, and photocarcinogenesis [2]. Upon acute exposure of the skin to UVB radiation, inflammatory responses to the degenerative processes are mediated primarily by the overproduction of intracellular reactive oxygen species (ROS) and damage to antioxidant defenses [3]. ROS, such as hydrogen peroxide and superoxide anions, induce DNA damage, inflammatory reactions, and damage to extracellular matrix (ECM) integrity [4].

Skin cells undergoing UVB-related senescence are characterized by increased levels of ROS, as well as hyperactivity of the transcription factor nuclear factor kappa B (NF-κB) and over-expression of inflammatory cytokines, such as tumor necrosis factor-alpha (TNF-α), cyclooxygenase-2 (COX-2), and interleukin 6 (IL-6). Therefore, chronic inflammatory responses due to chronic oxidative stress are a major risk factor for photoaging and other skin diseases [5,6]. UVB-induced ROS production triggers the expression of growth factor receptors, cell surface cytokines, and mitogen-activated protein kinases (MAPKs), such as c-Jun N-terminal kinase (JNK), extracellular signal-regulated kinase (ERK), and p38 kinase, which, in turn, regulate the activator protein 1 (AP-1) complex [7]. The transcriptional activity of the AP-1 complex, consisting of c-Jun and c-Fos, is determined by the degree of c-Jun phosphorylation and c-Fos expression [8]. Elevated AP-1 complex activity increases the production of matrix metalloproteinases (MMPs) and decreases the production of type I procollagen [9]. MMPs in the skin possesses collagenolytic activity and degrade ECM proteins, such as collagen, fibronectin, elastin, and proteoglycans, thus contributing to photoaging [10,11]. MMPs play an important role in photocarcinogenesis by regulating various processes related to tumor progression, including tumor establishment, growth, metastasis, and angiogenesis [12].

*Pinus densiflora* is widely consumed as a health supplement or food for health promotion and has recently been used as a cosmetic fuel [13]. *Pinus densiflora* contains numerous proanthocyanidins, which are a major class of polyphenols with potent antioxidant activity [14,15,16,17,18]. However, it is not known whether pine needle extracts protect against skin photoaging in human keratinocytes.

In this study, malonic acid (MA) isolated from *P. densiflora* was investigated to see if it affected UVB-induced expression of antioxidants and inflammation in HaCaT keratinocytes. In addition, it was confirmed that MA protected skin cells from UVB-induced photoaging by suppressing the underlying mechanisms of damage.

## 2. Materials and Methods

### 2.1. Chemical Reagents

Malonic acid (MA), 2′, 7′-dichlorodihydrofluorescein diacetate (DCF-DA), dimethyl sulfoxide, and epigallocatechin-3-gallate (EGCG), as a positive control of ROS induction, were purchased from Sigma-Aldrich (St. Louis, MO, USA). Fetal bovine serum (FBS), Dulbecco’s modified Eagle’s medium (DMEM), and trypsin-ethylenediaminetetraacetic acid were purchased from Welgene (Gyeongsan, Korea). SYBR green was purchased from GeneAll (Seoul, Korea). All antibodies (anti-heme oxygenase 1 (HO-1), anti-superoxide dismutase 1 (Sod1), anti-nuclear factor-erythroid 2-related factor-2 (Nrf2), anti-p-inhibitor of NF-κB (IκB), anti-IκB, anti-p-p65, anti-p65, anti-p50, anti-Cox-2, anti-IL-6, anti-TNF-α, anti-p-JNK, anti-JNK, anti-p-ERK, anti-ERK, anti-p-p38, anti-p38, anti-p-c-Jun, anti-c-Jun, anti-c-Fos, anti-p-Smad2/3, anti-Smad2/3, anti-collagen type I alpha 1 (Col1a1), anti-Col3a1, and anti-glyceraldehyde 3-phosphate dehydrogenase (Gapdh)) were purchased from Santa Cruz Biotechnology (Santa Cruz, CA, USA).

### 2.2. Plant Materials and Extraction of MA

**Figure 1:**
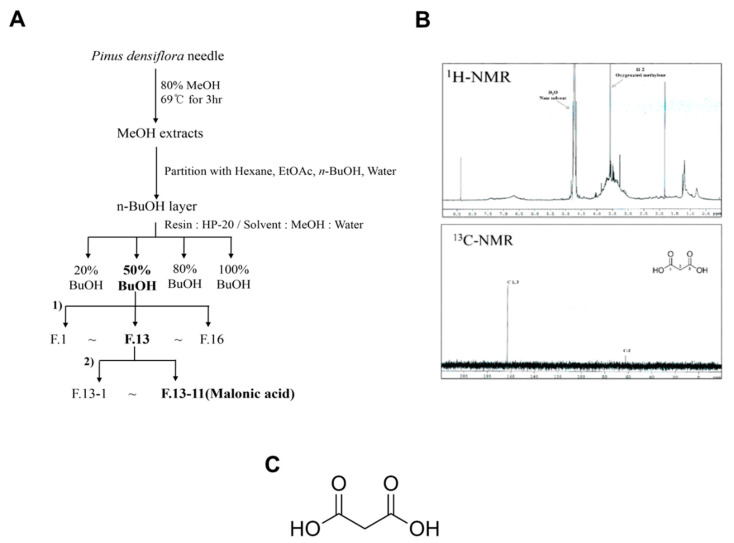
Isolation and purification of malonic acid (MA) from *P. densiflora.* (**A**) The *P. densiflora* n-BuOH layer has been separated and identified by nuclear magnetic resonance (NMR) to be MA. (**B**) 1H-NMR (300 MHz, D_2_O, δH) 3.45 (2H, s, H-2); 13C-NMR (75 MHz, D_2_O, δc) 167.5 (C-1,3), 62.2 (C-2). One methylene proton signal δH 3.45 (2H, s, H-2), two carboxyl δc 167.5 (C-1,3), and one methylene carbon signal δc 62.2 (C-2) in 1H- and 13C-NMR spectra suggest that it is MA. **(C)** Chemical structure of MA.

Fresh red pine (*P. densiflora Sieb. et Zucc*) needles were picked from a tree in Gokseong Province, Jeollanam-Do, South Korea. The harvested needles were cleaned with water (purified with 5% charcoal) and dehydrated using a spin-drier. The dried pine needles were used for extraction with 80% MeOH at 69 °C for 3 h. This crude extract was partitioned successively with n-hexane, EtOAc, n-BuOH, and H_2_O. The n-BuOH fraction, which exhibited strong antioxidant activity (Figure S1), was chromatographed over a silica gel column, which enabled the separation and isolation of its chemical constituents. The structures of the isolates were then analyzed by nuclear magnetic resonance (NMR) spectroscopy (Figure 1).

### 2.3. Cell Culture and UVB Irradiation

An immortalized human keratinocyte cell line, HaCaT, was obtained from the Anti-aging Research Institute of BIO-FD&C (Gwangju, Korea) and grown in DMEM containing 10% FBS and 100 µg/mL penicillin-streptomycin at 37 °C in a 5% CO_2_ humidified atmosphere. Cells were maintained until 80% confluence and then pretreated with various concentrations of MA. After a 4 h pretreatment, cells were washed with and covered with a thin coating of phosphate-buffered saline (PBS). Cells were then exposed to UVB light (15 mJ/cm^2^) for 60 s using a 280–315 mm UVB light source (CL-1000M UV Crosslinker; UVB-18, Upland, CA, USA). UV strength was measured using a UV radiometer (VLX-3.W; Vilber Lourmat, France). After UVB irradiation, the cells were treated with various concentrations of MA in a serum-free medium.

### 2.4. Cell Viability

HaCaT cell viability was determined using a cell proliferation assay (WST-1, Sigma-Aldrich, St. Louis, MO, USA). Cells cultured in 48-well plates (2 × 10^4^ cells/well) were pre-treated with MA (2 or 4 μM) for 4 h, washed with PBS, and then exposed to UVB (15 mJ/cm^2^) and MA treatment (2 or 4 μM) overnight in a serum-free medium. After treatment, 20 µL WST-1 assay reagent was added to each well, and the cells were then incubated for 30 min at 37 °C. After incubation, the absorbance of formazan products was measured at 450 nm using a multifunctional plate reader (Eon, BioTek, Winooski, VT, USA), and compared to untreated cells.

### 2.5. Evaluation of ROS Generation (DCF-DA Assay)

Intracellular ROS formation was assessed using DCF-DA as the substrate. First, HaCaT cells were seeded at 2 × 10^4^ cells/well in black 96-well plates. After 24 h, the cells were pretreated with MA (2 or 4 μM) for 4 h, washed with PBS, and subsequently exposed to UVB (15 mJ/cm^2^) and MA treatment (2 or 4 μM) overnight in a serum-free medium. Afterward, the cells were incubated with DCF-DA (20 µM) for 30 min. The formation of DCF due to the oxidation of DCF-DA in the presence of ROS was measured at excitation/emission wavelengths of 485/525 nm using a Gemini microplate reader (Molecular Devices, Sunnyvale, CA, USA).

### 2.6. RNA Isolation, cDNA Synthesis, and Real-time Quantitative Polymerase Chain Reaction (PCR)

Total RNA was isolated from cells using the Hybrid-R™ kit (GeneAll), according to the supplier’s instructions. To synthesize cDNA, 0.5 μM total RNA was primed with oligo dT and reverse transcribed using a cDNA synthesis HyperScript™ kit (GeneAll). cDNA was amplified using the RealAmp SYBR qPCR Master mix (GeneAll) containing specific primer pairs (Macrogen, Seoul, Korea). The reaction was performed at 95 °C for 10 min, followed by 40 cycles of amplification (95 °C for 10 s, 58 °C for 15 s, and 72 °C for 20 s). mRNA levels of specific genes were normalized to those of of Glyceraldehyde-3-Phosphate Dehydrogenase (GAPDH). The primer sequences of specific genes are described in Table S1. Amplification was measured using a Rotor-Gene RG-300 (Corbett, Hilden, Germany) instrument.

### 2.7. Western Blot Analysis

HaCaT cells were lysed in radioimmunoprecipitation assay buffer containing protease inhibitors and then incubated on ice for 10 min. Protein concentrations of cell lysates were determined using a BCA protein assay kit (Thermo, Rockford, IL, USA). Equal amounts of protein (20 μg) from each sample were loaded and separated by sodium dodecyl sulfate-polyacrylamide gel electrophoresis, and then transferred to a polyvinylidene difluoride membrane. The membrane was washed in Tris-buffered saline-0.1% Tween^®^ 20 (TBS-T) buffer (20 mM Tris-HCl, pH 7.5, 0.1% Tween 20), and non-specific binding sites were blocked using a blocking reagent (5% skim milk in TBS-T) for 1 h at room temperature on a shaker. The membrane was incubated overnight at 4 °C with the primary antibody in the blocking solution. After incubation, the membrane was washed in TBS-T buffer containing a diluted peroxidase-conjugated anti-mouse secondary antibody for 2 h at room temperature and washed again. The bound antibody was detected using a West-Zolplus detection reagent (iNtRON, Seonnam, Korea) and visualized on X-ray film.

### 2.8. Statistical Analysis

Statistical analyses were performed using GraphPad Prism software (version 5.0, GraphPad Software, San Diego, CA, USA). A significant difference between groups was evaluated using Student’s *t*-test. Values of *p* ˂ 0.05 were considered significant.

## 3. Results

### 3.1. Effects of MA on Oxidative Stress and Viability in UVB-induced HaCaT Cells

**Figure 2:**
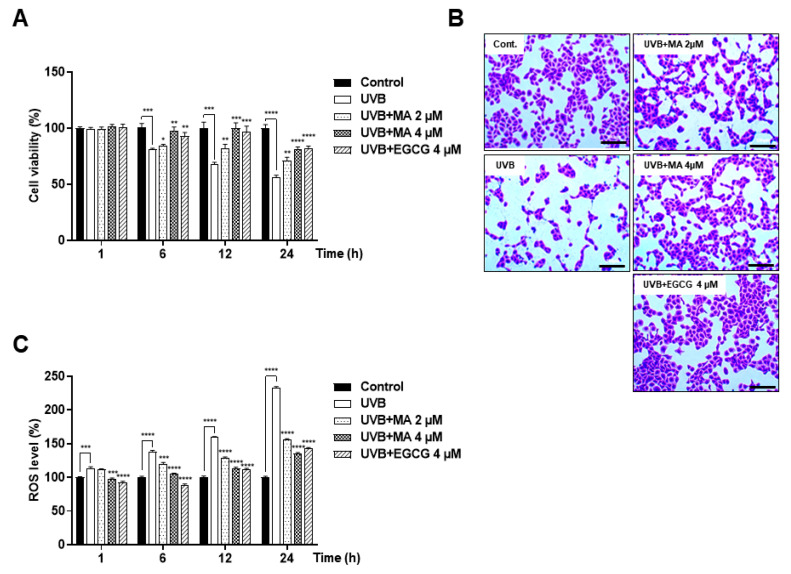
Effects of malonic acid (MA) on reactive oxygen species (ROS) levels and viability of ultraviolet (UV) B-irradiated HaCaT cells. HaCaT cells have been pre-treated with 2 or 4 μM MA and 4 μM epigallocatechin-3-gallate (EGCG) for 4 h and then exposed to UV-B irradiation (15 mJ/cm^2^). After UVB irradiation, cells have been incubated for 1, 6, 12, and 24 h. (**A**,**B**) Protective effects of MA against UVB-induced cell death, as measured by WST-1 assay and crystal violet staining. Scale bars, 100 µm. (**C**) Intracellular ROS levels induced by UVB-irradiation are measured using the 2′, 7′-dichlorodihydrofluorescein diacetate (DCF-DA) method. The data were analyzed in triplicate and displayed as the mean ± the standard error of the mean (SEM). (*) *p* < 0.05, (**) *p* < 0.01, (***) *p* < 0.001, and (****) *p* < 0.0001.

HaCaT cell viability was evaluated via WST-1 assay and crystal violet staining (Figure 2A,B). Moreover, the potential effect of MA on UVB-induced intracellular ROS generation was assessed using the DCF-DA assay (Figure 2C). HaCaT cells were treated with MA for 4 h prior to UVB exposure (15 mJ/cm^2^ for 1, 6, 12, and 24 h). These data showed that MA reduced UVB-induced ROS levels over time and inhibited ROS-mediated cell death in a dose-dependent manner.

### 3.2. Effects of MA on UVB-induced Antioxidant Enzyme Expression through Activation of Nrf2 in HaCaT Cells

**Figure 3:**
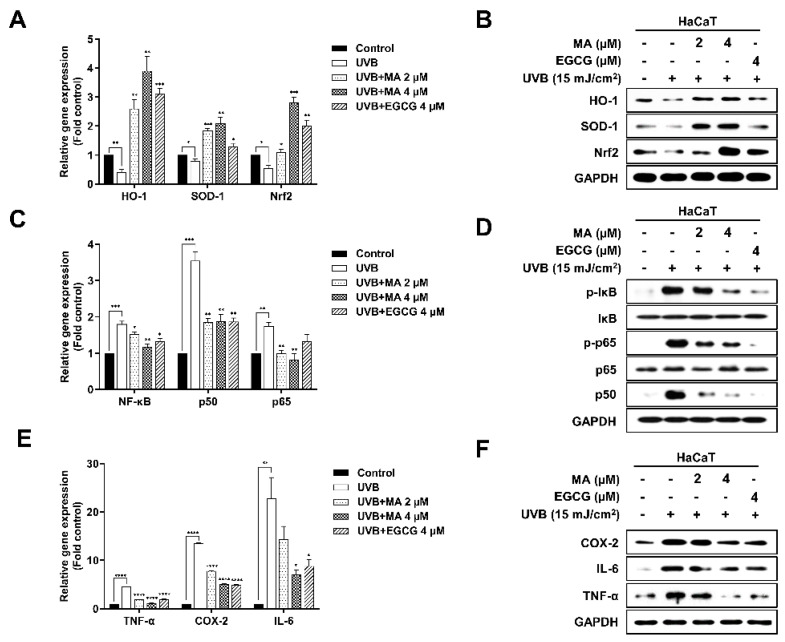
Effect of malonic acid (MA) on antioxidant enzymes, nuclear factor (NF)-κB activation, and inflammatory mediators in ultraviolet (UV) B-irradiated HaCaT cells. (A-F) HaCaT cells have been treated with MA for 4 h and then exposed to UVB irradiation (15 mJ/cm2). After an overnight incubation following UV exposure, the antioxidant enzymes (heme oxygenase 1 (HO-1), superoxide dismutase 1 (SOD-1), and nuclear factor-erythroid 2-related factor-2 (Nrf2)) are evaluated by (**A**) real-time polymerase chain reaction (PCR) and (**B**) western blot analysis. NF-κB, p50, and p65 are evaluated by (**C**) real-time PCR and (**D**) western blot analysis. The inflammatory mediators (tumor necrosis factor (TNF)-α, cyclooxygenase-2 (COX-2), and interleukin-6 (IL-6)) are evaluated by (**E**) real-time PCR and (**F**) western blot analysis. +, - means UVB irradiation or non-irradiation and treatment or non-treatment of MA and EGCG. Glyceraldehyde 3-phosphate dehydrogenase (GAPDH) is used as an internal control for western blot analysis. Epigallocatechin-3-gallate (EGCG) is used as a positive control. The data were analyzed in triplicate and displayed as the mean ± the standard error of the mean (SEM). (*) *p* < 0.05, (**) *p* < 0.01, (***) *p* < 0.001, and (****) *p* < 0.0001.

Nrf2 and Kelch-like ECH-associated protein 1 (Keap1) played an important role in regulating the transcriptional induction of antioxidant metabolic enzymes [19]. Therefore, the Nrf2-Keap1 pathway protected against photoaging by maintaining high levels of antioxidants [20]. In addition, Nrf2 regulation increased the expression of antioxidant enzymes, such as superoxide dismutase 1 (SOD-1) and heme oxygenase 1 (HO-1), making it an integral part of the defense system against ROS [21]. Therefore, Nrf2 activation, SOD-1, HO-1, and expression of antioxidant enzymes were confirmed using real-time PCR and western blot analysis (Figure 3A,B). mRNA expression of *nrf2*, *sod1*, and HO-1 in MA-treated HaCaT cells was higher than that in UVB-irradiated control cells (Figure 3A). Moreover, MA increased Sod-1, HO-1, and Nrf2 protein expression levels compared to that of the positive EGCG control group, as shown by western blot analysis (Figure 3B). These results showed that MA treatment inhibited ROS levels by activating Nrf2. Through these data, it was confirmed that MA inhibited intracellular ROS levels via Nrf2 activation and the expression of antioxidant enzymes.

### 3.3. Effects of MA on UVB-induced NF-κB Activation and Proinflammatory Factors in HaCaT Cells

Next, the effect of MA on UVB-induced expression of inflammatory mediators was investigated. MA treatment markedly suppressed the elements of the UVB-induced NF-κB signaling pathway, such as p65 and p50 (Figure 3C,D). Moreover, transcriptional activity and protein phosphorylation levels were lower than those in UVB-irradiated control cells. Furthermore, MA inhibited the expression of the inflammatory factors, TNF-α, COX-2, IL-6, IL-1β, and IL-8, in a dose-dependent manner in UVB-induced HaCaT cells (Figure 3E,F, Figure S2). These results demonstrated that MA suppressed UVB-induced expression of proinflammatory cytokines and the associated risk of skin cell damage.

### 3.4. Effects of MA on Phosphorylation during MAPK/AP-1signaling and MMP Expression in UVB-Induced HaCaT Cells

**Figure 4:**
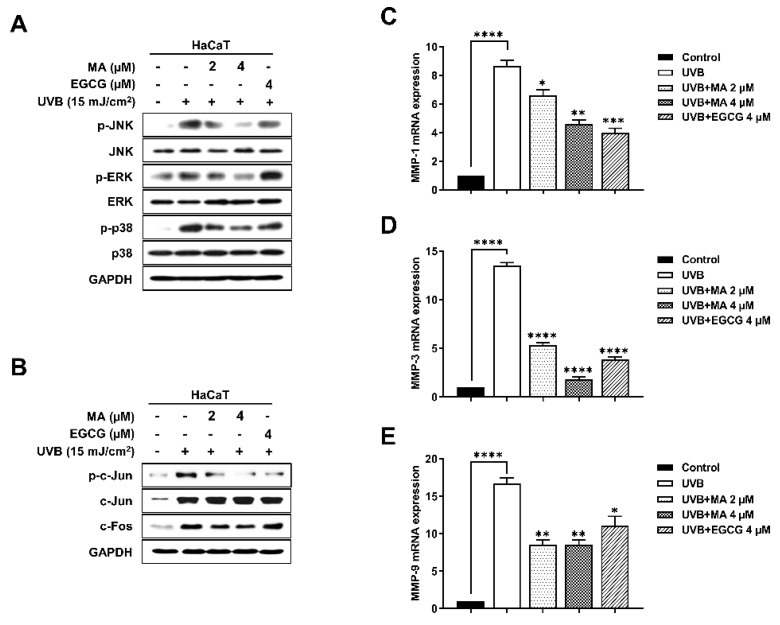
Effect of malonic acid (MA) on ultraviolet (UV) B-induced mitogen-activated protein kinase/activator protein 1 (MAPK/AP-1) signaling and matrix metalloproteinase (MMP) mRNA expression in UVB-irradiated HaCaT cells. (**A**–**E**) HaCaT cells have been treated with MA for 4 h and then exposed to UVB irradiation (15 mJ/cm^2^). After an overnight incubation following UV exposure, phosphorylation of (**A**) MAPK signaling (extracellular signal-regulated kinase [ERK], c-Jun N-terminal kinase [JNK], and p38) and (**B**) AP-1 complex (c-Jun and c-Fos) are assessed using western blot analysis. Glyceraldehyde 3-phosphate dehydrogenase (GAPDH) is used as an internal control for western blot analysis. Epigallocatechin-3-gallate (EGCG) is used as a positive control. (**C**–**E**) UVB-induced MMP expression in HaCaT cells. The expression of *mmp-1* (**C**), *mmp*-3 (**D**), and *mmp-9* (**E**) mRNA, as measured using real-time polymerase chain reaction (PCR). +, - means UVB irradiation or non-irradiation and treatment or non-treatment of MA and EGCG. The data were analyzed in triplicate and displayed as the mean ± the standard error of the mean (SEM). (*) *p* < 0.05, (**) *p* < 0.01, (***) *p* < 0.001, and (****) *p* < 0.0001.

UVB induces oxidative stress leading to phosphorylation via the MAPK signaling pathway [22]. The inhibitory effects of MA on JNK, ERK, and p38 phosphorylation were examined in HaCaT cells. MA treatment inhibited JNK, ERK, and p38 phosphorylation in a dose-dependent manner, compared to that of the UVB-irradiated control (Figure 4A). Thus, MA reduced phosphorylation of the AP-1 signaling pathway by suppressing MAPK signaling. AP-1 signaling involved the phosphorylation of c-Jun and c-Fos proteins, which were transcribed in the nucleus, and resulted in MMP expression and skin collagenolytic activity [23]. MA inhibited the AP-1 transcription factor, and the phosphorylation and protein levels of c-Jun and c-Fos (Figure 4B). The MAPK/AP-1 signaling pathway upregulated the mRNA expression of collagen-degrading MMPs, including MMP-1, MMP-3, and MMP-9, in HaCaT cells. MMPs were a crucial component of the pathophysiological photoaging mechanisms in the skin, and natural biomaterials that inhibit MMP expression in UV-damaged skin, successfully prevented photoaging [22]. Therefore, *mmp-1*, *mmp-3*, and *mmp-9* mRNA expression was confirmed using real-time PCR (Figure 4C–E). The PCR results showed that MA decreased the expression of MMPs by mediating the MAPK/AP-1 signaling pathway and inflammation. Thus, MA mediated its anti-photoaging activity by inhibiting MMP expression.

### 3.5. Effects of MA on the Transforming Growth Factor-β (TGF-β) Signaling Pathway and Collagen Synthesis Factors in UVB-Induced HaCaT Cells

**Figure 5:**
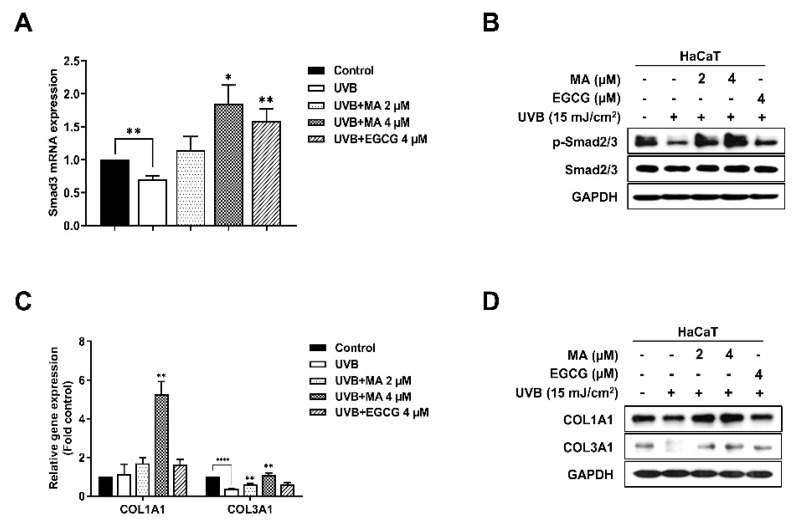
Effect of malonic acid (MA) on the expression of transforming growth factor (TGF)-β/Smad signaling components, collagen synthesis factor, and type I procollagen mRNA in ultraviolet (UV) B-irradiated HaCaT cells. (A-D) HaCaT cells have been treated with MA for 4 h and then exposed to UVB irradiation (15 mJ/cm^2^). After an overnight incubation following UV exposure, (**A**) *Smad3* mRNA expression is evaluated by real-time polymerase chain reaction (PCR), and (**B**) the protein level of phosphorylated Smad2/3 is measured by western blot analysis. (**C**) *col1a1* and *col3a1* mRNA expression are evaluated by real-time PCR, and (**D**) the protein levels of Col1a1 and Col3a1 are measured using western blot analysis. Glyceraldehyde 3-phosphate dehydrogenase (GAPDH) is used as an internal control for western blot analysis. Epigallocatechin-3-gallate (EGCG) is used as a positive control. +, - means UVB irradiation or non-irradiation and treatment or non-treatment of MA and EGCG. The data were analyzed in triplicate and displayed as the mean ± the standard error of the mean (SEM). (*) *p* < 0.05, (**) *p* < 0.01, (***) *p* < 0.001, and (****) *p* < 0.0001.

The TGF-β signaling pathway stimulates the growth of human dermal fibroblasts and induces the synthesis and secretion of major ECM protein collagen-growth factors, such as type I procollagen, COL1A1, and COL3A1 [24]. In addition, phosphorylated smad2/3 is a potent regulator of TGF-β signaling pathway activation [25,26]. Therefore, it was confirmed that UVB radiation regulates ECM tissue development and metabolism by modifying the TGF-β signaling pathway, thereby inhibiting collagen synthesis and promoting photoaging [27]. As mentioned above, TGF-β/Smad signaling promotes collagen synthesis and skin elasticity [28]. UVB-induced oxidative stress suppressed TGF-β/Smad signaling, thus downregulating collagen synthesis. To determine whether MA increased the expression of collagen synthesis factor by blocking the effects of UVB-irradiation on the TGF-β/Smad pathway, the mRNA and protein expression levels of Smad3 in HaCaT cells treated with UVB or MA were determined (Figure 5A,B). These data showed that MA promoted Smad3 expression and activity of Smad2/3 through phosphorylation compared to that of the UVB-irradiated control (Figure 5A,B). In addition, MA (2 or 4 μM) considerably increased mRNA expression and protein levels of COL1A1 and COL3A1 in HaCaT cells compared to that of the UVB-irradiated control (Figure 5C,D). Taken together, MA induced collagen growth by activating the TGF-β signaling pathway and through Smad2/3 activity, which was inhibited by UVB-induced ROS.

## 4. Discussion

*P. densiflora* leaves have been used in traditional medicine for the treatment of hemorrhages, gastroenteric disorders, hypertension, asthma, and liver and skin diseases [29]. While the chemicals in *P. densiflora* have antioxidant and antibacterial activities [30,31], they have rarely been investigated in relation to UVB-induced skin inflammation and photoaging. Therefore, the protective effect of MA against photoaging and skin inflammation was investigated, as well as the underlying molecular mechanisms.

UVB radiation is a potent environmental risk factor for skin cancer and photoaging [32,33,34]. When the skin is irradiated by UVB, ROS production, one of the first events to occur in skin cells, induces signaling pathways for inflammatory processes and other biochemical reactions related to oxidative cell damage [35]. In particular, UVB-induced ROS production also upregulates the expression of MMPs, which enhance collagen breakdown in the dermis, leading to increased collagen degradation [36,37]. As a result, ROS are generally considered to be a major cause of photoaging [38]. Thus, reducing ROS generation is a potentially effective strategy to prevent skin photoaging.

**Figure 6:**
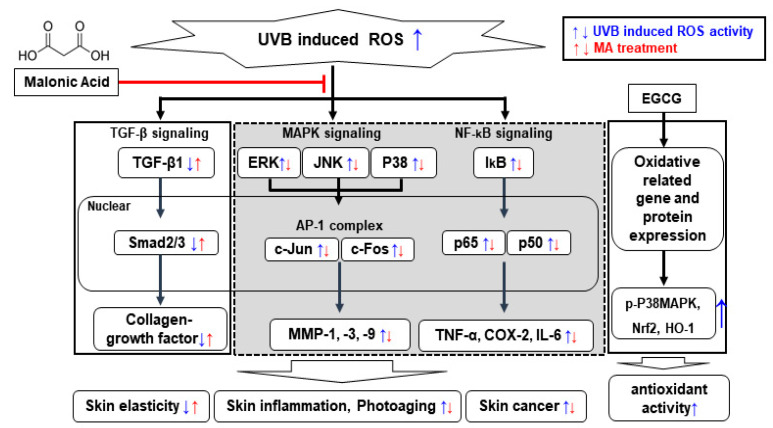
A schematic model of malonic acid (MA) inhibiting ultraviolet (UV) B-induced reactive oxygen species (ROS) activity**.** MA inhibits UVB-induced ROS in skin cells and suppresses mitogen-activated protein kinase (MAPK) and nuclear factor (NF)-κB signaling pathways through the reduced activity of the MAPK/activator protein 1 (AP-1) complex, matrix metalloproteinase (MMP) expression, and NF-κB activity. In addition, the transforming growth factor (TGF)-β/Smad signaling pathway is increased to promote collagen production. These results suggest MA inhibits skin inflammation and photoaging. (Blue arrow: ROS induced signaling without MA treatment, Red arrow: ROS signaling with MA treatment).

In this study, it was demonstrated that MA protected HaCaT keratinocytes by reducing the amount of ROS generated by UVB irradiation. Convincing evidence was also provided that MA played an important role in inhibiting the ROS signaling pathway (Figure 6).

At first, MA significantly decreased UVB-induced intracellular ROS production and increased cell survival rate by increasing antioxidant enzyme expression. Antioxidant enzymes mediate decreased ROS production and protect the skin from the effects of UV irradiation [20]. The Nrf2-Keap1 pathway plays a key role in regulating the transcriptional induction of various antioxidant metabolizing enzymes, such as SOD-1 and HO-1, which neutralize the damaging effects of ROS [20].

Second, MA attenuated UVB-induced proinflammatory cytokine expression and protein levels. The NF-κB signaling pathway is an important regulator of gene expression associated with cell proliferation, immune response, and inflammatory factors [6]. In addition, NF-κB increases MMP expression in the dermis [39]. These results showed that MA reduced the expression levels of the transcription factors IκB, p65, and p50 and inflammatory cytokine factors, such as TNF-α, COX-2, IL-6, IL-1β, and IL-8. These reduced genes and the NF-κB signaling pathway affected MMP expression, photoaging, and skin cancer. The antioxidant properties of MA were confirmed by its ability to increase antioxidant enzyme activity under oxidative stress conditions. Therefore, MA significantly inhibited inflammatory cytokine expression via these factors.

In addition, MA inhibited the MAPK/AP-1 signaling pathway, thereby attenuating the expression of MMPs and ultimately providing protection against photoaging [22]. The MAPK signaling pathway plays an important role in regulating cell proliferation, cell motility, MMP gene expression, and photoaging. Mammalian cells have three groups of MAPKs: JNK, ERK, and p38 [40]; ROS-induced phosphorylation of JNK, ERK, and p38 results in the expression of the AP-1 signaling pathway. Increased AP-1 signaling and the phosphorylation of c-Jun and c-Fos upregulate MMPs while downregulating type I procollagen [38]. Here, the UVB-induced phosphorylation of ERK, JNK, and p38 decreased in MA-treated HaCaT cells. Additionally, MA suppressed AP-1 activation by reducing c-Jun phosphorylation and c-Fos expression.

Finally, MA regulated the TGF-β signaling pathway by regulating Smad2/3 expression. Furthermore, MA increased COL1A1 and COL3A1 mRNA expression and protein levels. TGF-β exhibits various biological activities, such as ECM deposition and tissue regeneration, cell attachment and proliferation, and inflammatory response in wounded areas [41]. TGF-β binds to the TGF-β receptor complex and activates phosphorylated Smad2/3, which induces the expression of the target gene, thus regulating cell function [28]. Smad2/3 conveys the signal from cell-surface receptors to the procollagen gene promoter in human dermal fibroblasts [42,43]. TGF-β enhances the wound-healing process via induction and deposition of ECM molecules, including COL1A1 and COL3A1. However, UVB-irradiation inhibits collagen synthesis by altering the TGF-β/Smad signaling pathway [26]. Here, it was shown that MA restores Smad2/3 mRNA expression and phosphorylated protein levels in UVB-irradiated HaCaT cells.

## 5. Conclusions

These results demonstrated that MA inhibited UVB-induced oxidative stress and inflammation via reduced expression of antioxidant enzymes, MMPs, and proinflammatory cytokines. Inhibition of MMP expression reduced collagen degradation and protected against ECM damage. Moreover, MA inhibits inflammatory factor activity. Overall, these results suggested that MA acts as a skin protectant against UVB-induced photoaging and skin inflammatory diseases. Furthermore, MA will be available as a pharmaceutical product.

## Data Availability

The data presented in this study are available on request from the corresponding author.

## Supplementary Materials

The following are available online at https://www.mdpi.com/2073-4360/13/5/816/s1, Figure S1: The 50% n-BuOH fraction, which exhibits strong antioxidant activity. Figure S2: Effects of malonic acid (MA) on ultraviolet (UV) B-induced proinflammatory mediator expression levels in HaCaT cells. Table S1: Primer lists
