# Supplementary material for: Malonic Acid Isolated from Pinus densiflora Inhibits UVB-Induced Oxidative Stress and Inflammation in HaCaT Keratinocytes
Source: Polymers (Basel). 2021 Mar 7;13(5):816. doi: 10.3390/polym13050816 (PMC7961482; doi:10.3390/polym13050816)
Supplement: Supplementary file 1 [file polymers-13-00816-s001.pdf]

Supplementary Information

Supplementary Table S1. Primer lists

Genes		sequences
**Nrf-2**	**Sense**<br>**Anti-sense**	5'- CAG CGA CCT TCG CAA ACA AC -3'<br>5'- CAT GAT GAG CTG TGG ACC GT -3'
**SOD-1**	**Sense**<br>**Anti-sense**	5'- ATG GCG ACG AAG GCC GTG TG-3'<br>5'- GAC CAC CAG TGT GCG GCC AA-3'
**HO-1**	**Sense**<br>**Anti-sense**	5'- AGG GAA TTC TCT TGG CTG GC-3'<br>5'- GAC AGC TGC CAC ATT AGG GT-3'
**MMP-1**	**Sense**<br>**Anti-sense**	5'- TCT TGG ACT CTC CCA TTC TAC T-3'<br>5'- TAG CTT ACT GTC ACA CGC TTT T-3'
**MMP-3**	**Sense**<br>**Anti-sense**	5'- TGA GGA CAC CAG CAT GAA CC-3'<br>5'- ACT TCG GGA TGC CAG GAA AG-3'
**MMP-9**	**Sense**<br>**Anti-sense**	5'- GGG CCG CTC CTA CTC TGC CT-3'<br>5'- TCG AGT CAG CTC GGG TCG GG-3'
**NF-kB**	**Sense**<br>**Anti-sense**	5'- TCT TGG ACT CTC CCA TTC TAC T-3'<br>5'- TAG CTT ACT GTC ACA CGC TTT T-3'
**P65**	**Sense**<br>**Anti-sense**	5'- TGA GGA CAC CAG CAT GAA CC -3'<br>5'- ACT TCG GGA TGC CAG GAA AG-3'
**P50**	**Sense**<br>**Anti-sense**	5'- GGG CCG CTC CTA CTC TGC CT-3'<br>5'- TCG AGT CAG CTC GGG TCG GG-3'
**TNF-α**	**Sense**<br>**Anti-sense**	5'- AGC TCC CTC TAT TTA TGT TTG C-3'<br>5'- TAC ATG GGA ACA GCC TAT TGT-3'
**COX-2**	**Sense**<br>**Anti-sense**	5'- TCC CAC AGT CAA AGA TAC TCA G-3'<br>5'- CTG TTT AAG CAC ATC GCA TAC T-3'
**IL-6**	**Sense**<br>**Anti-sense**	5'- TCG AGC CCA CCG GGA ACG AAA-3'<br>5'- AGG CAA CTG GAC CGA AGG CG-3'
**IL-8**	**Sense**<br>**Anti-sense**	5'- CCC CAA TAA ATA TAG GAC TGG A-3'<br>5'- GCT GCT TTC ACA CAT GTT ACT C-3'
**IL-1β**	**Sense**<br>**Anti-sense**	5'- CAC CTC TCC TAC TCA CTT AAA GC-3'<br>5'- GGC TCT TTT ACA GAC ACT GCT AC-3'
**Smad3**	**Sense**<br>**Anti-sense**	5'-GGC TGC TCT CCA ATG TCA -3'<br>5'- CAC TCT GCG AAG ACC TCC-3'
**COL1A1**	**Sense**<br>**Anti-sense**	5'-TCT GAC TGG AAG AGT GGA GAG TA -3'<br>5'- GTT CTT GCT GAT GTA CCA GTT CT-3'
**COL3A1**	**Sense**<br>**Anti-sense**	5'- CCT GAA CTC AAG AGT GGA GAA TA-3'<br>5'- CTA GAA TCT GTC CAC CAG TGT TT-3'
**GAPDH**	**Sense**<br>**Anti-sense**	5'- TCA ACG GAT TTG GTC GTA TT-3'<br>5'- GGA TTT CCA TTG ATG ACA AG-3'

Supplementary Figure legends

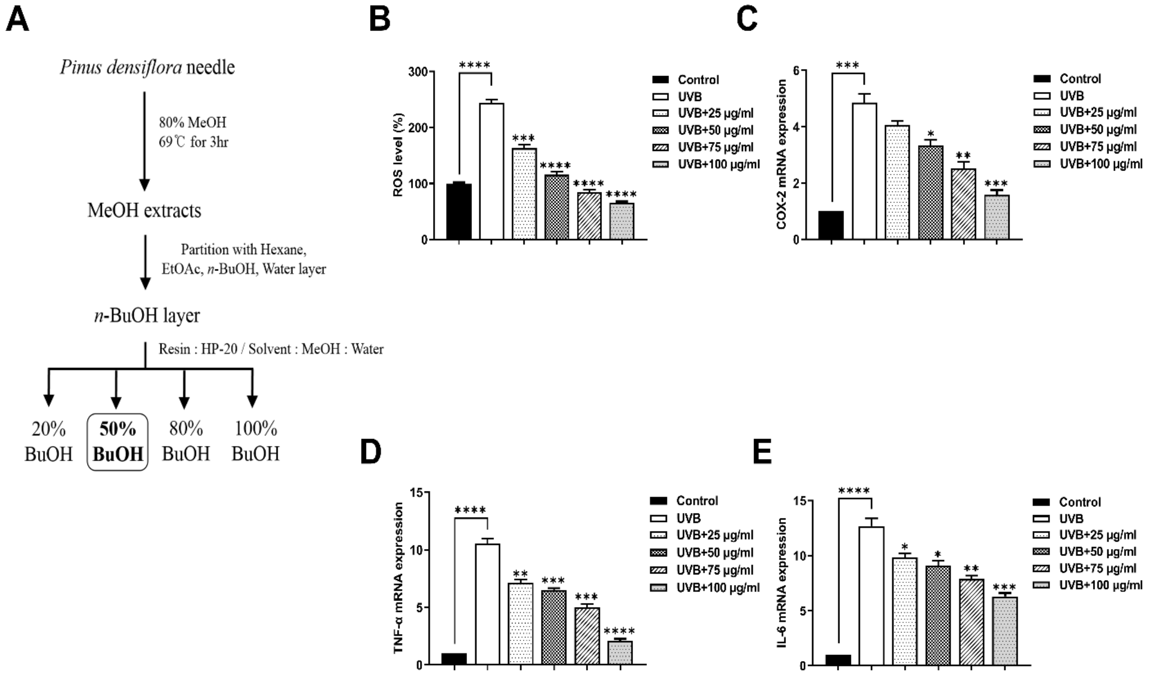

Supplementary Figure S1. The 50% n-BuOH fraction, which exhibits strong antioxidant activity. (A) n-BuOH layer fractionated using HP-20 column chromatography and a gradient of MeOH:water (from 20:80 to 100:0), to yield four combined fractions. **(B-E)** HaCaT cells are pretreated for 4 h with the 50% BuOH layer and then irradiated with ultraviolet (UV) B (15 mJ/cm2). **(B)** The levels of reactive oxygen species (ROS) are estimated indirectly by measuring the fluorescence emitted by 2', 7'-dichlorodihydrofluorescein diacetate (DCF-DA)**, (C-E)** mRNA expression of cyclooxygenase-2 (*cox-2*), tumor necrosis factor-alpha (TNF-*α*) and interleukin-6 (*il-6*) are evaluated by real-time polymerase chain reaction (PCR). Data are expressed as the mean ± the standard error of the mean (SEM). (*) $P < 0.05$, (**) $P < 0.01$, (***) $P < 0.001$, and (****) $P < 0.0001$.

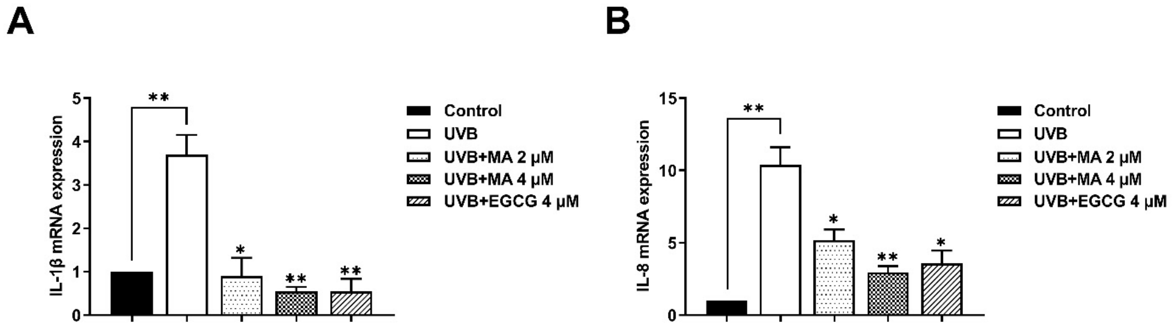

Supplementary Figure S2 (related to Figure 3C). Effects of malonic acid (MA) on ultraviolet (UV) B-induced proinflammatory mediator expression levels in HaCaT cells. (A-B) HaCaT cells are pretreated with MA for 4 h and then exposed to UVB irradiation (15 mJ/cm2). After overnight UVB exposure, interleukin-1β (*il-1β*) **(A)** and *il-8* **(B)** expression are evaluated by real-time polymerase chain reaction (PCR). Data are expressed as the mean ± the standard error of the mean (SEM). (*) $P < 0.05$, (**) $P < 0.01$, (***) $P < 0.001$, and (****) $P < 0.0001$.
